# Paralysis, Colic, &c. Connected with Intestinal Worms

**Published:** 1829-10-01

**Authors:** 


### HOSPICE DE LA SALPETRIERE.

PARALYSIS, COLIC, VIOLENT HEAD-ACHE, &c. CONNECTED WITH INTESTINAL WORMS.*

Every medical man of experience must have witnessed the frequent connexion of chorea and other convulsive affections with intestinal irritation, and particularly worms. These are more frequent in adults than is commonly imagined, and often prove a stumbling block to our morbid anatomists, who look for disorganizations and destructions in every case of head-ache or paralysis. The following short narrative may prove instructive to these ultra pathologists, or rather DISORGANISTS, for pathology is quite another thing.

Mademoiselle Niguet, a pauper of the Salpêtrière, was first attacked twenty years ago with violent colic and tenesmus, for which she could assign no cause. A purgative which was administered produced some copious stools and relieved the symptoms, but they soon returned with increased severity and baffled every means employed against them. In the midst of one of the colicky paroxysms, accompanied with violent contractions of the abdominal muscles, the left leg was suddenly struck with palsy, of which she still retains the traces. About this time also she began to suffer from permanent pain in the head and lumbar region, which disappeared or diminished when the colics returned. This was every month at the period of the catamenia, which, however, were perfectly regular, and the violent pains and tenesmus were only relieved after very abundant bilious evacuations. When her distressing complaint had been established for

* Journ. Hebdom. No. 44.

some time she grew sallow, anxious, and desponding; her respiration was interrupted by a small dry cough; her pulse was feeble; her tongue white; her digestion extremely bad. Her existence, in fact, was rendered miserable by headaches, loss of appetite, thirst, constipation, and a paroxysm of colic every month.

In 1824, whilst labouring under one of these attacks of colic and tenesmus, Niguet passed by stool a worm which she compared to the bone of the ray fish, about two lines in breadth, marked transversely, and nearly half an ell in length. Abundant bilious dejections accompanied the expulsion of the worm, and the pains subsided. They soon, however, reappeared, and terminated as before by the evacuation of bilious matters, in the midst of which swarmed many small living worms, nearly an inch in length, and marked like the first. In 1825 the patient voided, under similar circumstances, another flat worm of extraordinary length, and a third made its appearance in 1826, about six or seven inches long. She now consulted a pupil of the Salpetrière, who gave her a decoction of the rind of the pomegranate, which she took, though irregularly, for a long time. No relief was obtained, and the patient being again seized in January, 1828, with the most insupportable colic, and voiding a fourth worm three quarters of an ell in length, she entered the Infirmary, where the Indian method, *methode Indienne*, was had recourse to. The attacks of colic were by this means warded off for some time, but they have since re-appeared, and so have the other symptoms together with very fatiguing vomiting every morning.

Whether the colic was, in the first instance, produced by the lurking tænia, or the tænia by the long continued colic and indigestion, is a question that is not to be readily solved. Worms will remain long dormant, or at least produce no decided symptoms; but on the other hand, indigestion and morbid secretions will beyond a doubt predispose to their occurrence. At all events the preceding case displays the heterogeneous and violent symptoms to which these parasitic animals, when once established in the intestinal canal, will give rise.

